# Special Issue: Cancer Metabolism

**DOI:** 10.3390/metabo7030041

**Published:** 2017-08-09

**Authors:** Madhu Basetti

**Affiliations:** Imaging Core, Cancer Research UK Cambridge Institute, University of Cambridge, Cambridge CB2 0RE, UK; Madhu.Basetti@cruk.cam.ac.uk; Tel.: +44-1223-769645; Fax: +44-1223-769510

**Keywords:** cancer metabolism, Warburg effect, oncogenes, onco-metabolite, glioblastoma, paraganglioma, tumour suppressor genes, chemotherapy, NMR, MRI, metabolomics, p53

## Abstract

This special issue is designed to present the latest research findings and developments in the field of cancer metabolism. Cancer is a complex disease and a common term used for more than 100 diseases, whereas metabolism describes a labyrinth of complex biochemical pathways in the cell. It is essential to understand metabolism in the context of cancer for the early detection of disease biomarkers and to find proper targets for potential treatments. The articles presented in this issue cover metabolic aspects of brain tumours, breast tumours, paraganglioma, and the metabolic activity of tumour suppressor gene p53.

The genetic mutations that arise from aging, environmental exposure, hereditary background, or lifestyle are regarded as the main risk factors for cancer. More than 90 years of biochemical observations of cancer cells, tumour tissues, and organs with cancer all point to the fact that the malignant transformation of cells can cause metabolic reprogramming to sustain tumour growth.

In the 1920s, German scientist Warburg reported the abnormal metabolism of tumour cells that resulted in a shift from ATP generation through oxidative phosphorylation to ATP generation through glycolysis, even in the presence of oxygen. Despite this, over the past six decades, molecular biology has been the major focus of cancer research. The past decade has seen a revival of interest to better understand cancer metabolism and its association with oncogenes/tumour suppressor genes. Many links exist between metabolism and genetic mutations in cancer cells [1]. Oncogenes promote, whereas tumour suppressor genes inhibit carcinogenesis, while both regulate metabolism [1]. The recent interesting findings on glycolysis, glutaminolysis, serine/glycine metabolism, amino acid metabolism, lipid and membrane lipid metabolism, and TCA cycle enzyme (SDH, FH and IDH) mutations in cancer cells led to a renewed interest in the field of cancer metabolism. The emergence of systems biology, following the publication of the complete human genome, has focused on the integration of information from gene regulation with the molecular phenotypes of proteins (including enzymes) and metabolites. A more complete understanding of cancer metabolism will be essential to define the underlying cellular networks and proper targets for developing anti-cancer treatments.

In this special issue, Professor Schulze’s group thoroughly reviewed the regulation of metabolic activity by tumour suppressor gene p53 [2]. Tumour suppressor p53, regarded as the master guardian of the genome, was found to be inactivated in 50% of all human tumours. The p53 regulation of glycolysis (ancient biochemical pathway for catabolism and anabolism) and mitochondria metabolism (the energy production unit in the cell), as well as one carbon metabolism (serine/glycine) and lipid metabolism through multiple mechanisms are presented in this review [2]. In addition, they also presented several aspects of the active role of p53 in immune regulation and tumour microenvironment in their review.

Professor Favier’s group presented the current view of how mitochondrial metabolic deficiencies may lead to cancer predisposition in paragangliomas [3]. The Warburg hypothesis, that mitochondrial defects are the main cause of tumorigenesis, received a boost from the findings of mutations in enzymes of the TCA cycle. In the last two decades, findings of mutations in enzymes Succinate dehydrogenase (SDH) [4,5], fumarate hydratase (FH) [6], and iso-citrate dehydrogenase (IDH) [7] reinforced the notion of a connection between mitochondrial defects and tumorigenesis. The tumours with SDH accumulate succinate while FH mutation will result in fumarate accumulation, which can be readily detected in in vivo systems with analytical methods such as NMR, due to their concentration levels being in the milli-molar range. Mutations in IDH lead to a neomorphic ability to produce an ‘onco-metabolite’ called 2-hydroxyglutarate (2HG), and can be detected in human brain tumours with clinical MRI scanners [8,9,10]. In another review, Lussey-Lepoutre et al. presented how these metabolic deficiencies of SDH and FH may lead to cancer predisposition and also showed that they may lead to the development of innovative therapeutic strategies and precision medicine approaches for the management of paraganglioma patients [3]. 

The MRS detection of 2HG as a biomarker for IDH mutation in gliomas was reviewed by Professor Poptani’s group [10]. As mentioned earlier, mutation in the IDH gene results in the gain of function for IDH enzyme, and this neomorphic enzyme produces 2HG from cancer cells in a detectable concentration by in vivo MRS methods using clinical MR scanners. Leather et al. reviewed their development in addition to the detailed presentation of the biochemistry of 2HG, its use as a biomarker, the prognostic impact of IDH mutation, and potential treatment advances in IDH mutant gliomas [10].

The research article in this special issue by Arias-Ramos et al. [11] focused on the non-invasive assessment of response to the temozolamide treatment of pre-clinical gliomas by using a multi-slice MRSI-based volumetric analysis. Gliomas are common aggressive brain tumours with a short survival time and limited therapy choices. These authors presented their work with the development of the tumour response index (TRI) to measure the response of the treatment from MRSI-based nosological images. Each pixel in the nosological image is coloured according to a class (e.g., tumour, necrosis, and normal tissue). The further validation of MRSI-observed results was conducted by the analysis of histological images [11].

Breast and prostate cancers have additional complication with their association with hormonal dependence. In this issue, two groups reviewed breast cancer metabolism from two different perspectives. Professor Bathen’s group approached the understanding of breast cancer tissue metabolism from a systems biology view point [12], while Professor Jagannathan’s group focused on the clinical applications of MRS in breast cancer [13]. Both groups overlap with their coverage of metabolic pathways. Integrating metabolomics data from cells and tissue samples with other omics (transcriptomics and proteomics) has been one of the main tasks in the last two decades to gain a clear understanding of the underlying biology of this human disease. 

Haukaas et al. [12] reviewed metabolomics data from HRMAS ^1^H NMR of ex vivo breast cancer tissues. They presented a detailed analysis of glycolysis, amino acid, and phospholipid metabolic pathways and their association with gene expressions of certain genes encoding the proteins of the concerned pathway. The review also covered metabolic aspects of the different sub-types of breast cancer, depending on the tissue type (basal and luminal) and hormonal dependence nature (estrogen receptor positive/negative, progesterone receptor positive/negative, and human epidermal growth factor receptor 2 (HER2/neu)).

The review of Jagannathan et al. [13] focused on the different NMR and MRS methodological aspects in obtaining metabolic information from cells, ex vivo biopsies, and in vivo human tissues in clinical settings. The methodologies and data included in this review ranged from in vitro HR-NMR and ex vivo HRMAS ^1^H NMR to in vivo MRS on a clinical MR scanner, with samples from cells, biopsies, and human patient subjects in clinical studies. Possibilities of new data for breast cancer from emerging methods such as hyperpolarised ^13^C NMR and data of breast tissues metabolism from the established method of ^31^P NMR are also presented in detail in this review [13].

In the last 100 years of biochemistry, several biochemical pathways have been discovered, and the Michaelis-Menten model accounts for the kinetic properties of many enzymes in these pathways. Several tracer studies in the 1960s (and also by recent hyperpolarised ^13^C NMR methods) probed these metabolic pathways by measuring their metabolic flux. In the last decade, there have been several new interesting findings that have changed the view of how a metabolic pathway function is regulated through gene-regulation, epigenetic modifications, metabolic environment, and the still-emerging field of microRNA interactions. A recent review outlined six major emerging hallmarks of cancer metabolism comprised of deregulated uptake of glucose and amino acids, opportunistic mode of nutrients acquisition, use of glycolysis/TCA cycle intermediates for biosynthesis, increased nitrogen demand, alterations in metabolite-driven gene regulation, and metabolic interactions with the tumour microenvironment [14]. In essence, the mutual interdependence of mutated gene regulation (as well as their products) and reprogrammed cancer cellular metabolism demands new ways of studying cancer metabolism. In this regard, I hope the abovementioned reviews and articles in this special issue will address some of the current research goals in the emerging field of cancer metabolism. 

To conclude, I would like to thank each of the authors for their contribution in producing this special issue on cancer metabolism.

Finally, I wish to acknowledge the thorough work of the peer reviewers who contributed to improving the submitted manuscripts and I also would like to thank the staff members of the *Metabolite* Editorial Office for their support.

## References

[1] Dang C.V. (2012). Links between metabolism and cancer. Genes Dev..

[2] Floter J., Kaymak I., Schulze A. (2017). Regulation of metabolic activity by p53. Metabolites.

[3] Lussey-Lepoutre C., Buffet A., Gimenez-Roqueplo A.P., Favier J. (2017). Mitochondrial deficiencies in the predisposition to paraganglioma. Metabolites.

[4] Baysal B.E., Ferrell R.E., Willett-Brozick J.E., Lawrence E.C., Myssiorek D., Bosch A., van der Mey A., Taschner P.E., Rubinstein W.S., Myers E.N. (2000). Mutations in *SDHD*, a mitochondrial complex II gene, in hereditary paraganglioma. Science.

[5] Astuti D., Latif F., Dallol A., Dahia P.L., Douglas F., George E., Skoldberg F., Husebye E.S., Eng C., Maher E.R. (2001). Gene mutations in the succinate dehydrogenase subunit SDHB cause susceptibility to familial pheochromocytoma and to familial paraganglioma. Am. J. Hum. Genet..

[6] Tomlinson I.P., Alam N.A., Rowan A.J., Barclay E., Jaeger E.E., Kelsell D., Leigh I., Gorman P., Lamlum H., Rahman S. (2002). Germline mutations in FH predispose to dominantly inherited uterine fibroids, skin leiomyomata and papillary renal cell cancer. Nat. Genet..

[7] Parsons D.W., Jones S., Zhang X., Lin J.C., Leary R.J., Angenendt P., Mankoo P., Carter H., Siu I.M., Gallia G.L. (2008). An integrated genomic analysis of human glioblastoma multiforme. Science.

[8] Andronesi O.C., Kim G.S., Gerstner E., Batchelor T., Tzika A.A., Fantin V.R., Vander Heiden M.G., Sorensen A.G. (2012). Detection of 2-hydroxyglutarate in *IDH*-mutated glioma patients by in vivo spectral-editing and 2D correlation magnetic resonance spectroscopy. Sci. Transl. Med..

[9] Choi C., Ganji S.K., DeBerardinis R.J., Hatanpaa K.J., Rakheja D., Kovacs Z., Yang X.L., Mashimo T., Raisanen J.M., Marin-Valencia I. (2012). 2-hydroxyglutarate detection by magnetic resonance spectroscopy in *IDH*-mutated patients with gliomas. Nat. Med..

[10] Leather T., Jenkinson M.D., Das K., Poptani H. (2017). Magnetic resonance spectroscopy for detection of 2-hydroxyglutarate as a biomarker for IDH mutation in gliomas. Metabolites.

[11] Arias-Ramos N., Ferrer-Font L., Lope-Piedrafita S., Mocioiu V., Julia-Sape M., Pumarola M., Arus C., Candiota A.P. (2017). Metabolomics of therapy response in preclinical glioblastoma: A multi-slice MRSI-based volumetric analysis for noninvasive assessment of temozolomide treatment. Metabolites.

[12] Haukaas T.H., Euceda L.R., Giskeodegard G.F., Bathen T.F. (2017). Metabolic portraits of breast cancer by HR MAS MR spectroscopy of intact tissue samples. Metabolites.

[13] Jagannathan N.R., Sharma U. (2017). Breast tissue metabolism by magnetic resonance spectroscopy. Metabolites.

[14] Pavlova N.N., Thompson C.B. (2016). The emerging hallmarks of cancer metabolism. Cell Metab..

